# Osteoarthritic Severity in Unresurfaced Patellae Does Not Adversely Affect Patient-reported Outcomes in Contemporary Primary TKA

**DOI:** 10.5435/JAAOSGlobal-D-22-00041

**Published:** 2022-04-06

**Authors:** Gregory J. Schmidt, Hassan Farooq, Evan R. Deckard, R. Michael Meneghini

**Affiliations:** From the Department of Orthopaedic Surgery, Indiana University School of Medicine, Indianapolis, IN (Dr. Schmidt, Farooq, Deckard, and Dr. Meneghini), and the IU Health Hip & Knee Center, IU Health Saxony Hospital, Fishers, IN (Dr. Meneghini).

## Abstract

**Introduction:**

The degree of osteoarthritis (OA) acceptable to leave in a native patella during unresurfaced total knee arthroplasty (TKA) remains unknown. This study's purpose was to examine the effect of patellofemoral OA severity on patient-reported outcome measures (PROMs) in primary TKAs performed without patellar resurfacing.

**Methods:**

One hundred ninety-three primary TKAs performed without patellar resurfacing were retrospectively reviewed. Preoperative patellofemoral OA severity was graded on severity, marginal osteophytes, joint space narrowing, and chondral damage using accepted grading systems. Patellar tilt and tibiofemoral alignment were measured radiographically. PROMs were evaluated at a minimum of 1-year follow-up.

**Results:**

In multivariate regression, preoperative lateral patella Kellgren-Lawrence grade ≥2 was associated with superior change in Knee Society Score pain with level walking, higher absolute change in Knee Injury and Osteoarthritis Outcome Score for Joint Replacement (*P* ≤ 0.029), and knees ‟always feeling normal” (odds ratio [OR] 3.12; *P* = 0.005). Osteoarthritis Research Society International atlas grades and Outerbridge classification scores did not significantly influence PROMs.

**Discussion:**

Worse preoperative OA severity in the lateral patellar facet, graded with the Kellgren-Lawrence system, predicted superior knee-specific PROMs in patients with unresurfaced patellae after contemporary TKA. This observation supports the clinical finding that patients with more severe OA have optimized patient outcomes and highlights the minimal contribution of patella OA to knee function after primary TKA.

Patellar resurfacing remains a controversial topic for the treatment of osteoarthritis (OA) in the patellofemoral joint (PFJ) during total knee arthroplasty (TKA).^1,2,3,4^ Currently, patellar resurfacing during primary TKA is the most popular technique in the United States according to the American Joint Replacement Registry report.^5^ However, complications related to costly patellar implant revisions persist.^4,6-8^ Given these potential risks associated with patellar resurfacing and the inconclusive nature of previous resurfacing studies questioning the causative etiology of anterior knee pain related to unresurfaced patellae, a shift toward leaving the patella unresurfaced during primary TKA has steadily increased from 4.1% in 2012 to 9.6% in 2020^5^ with acceptable outcomes up to 10 years.^9^ In addition, modern TKA implants are more “patella-friendly,” designed with a more anatomic trochlear groove to accommodate near-native patellofemoral tracking and forces.^2,10^

Previous reports demonstrate an increased risk of re-operation after TKA with unresurfaced patellae mostly because of persistent anterior knee pain.^11,12,13,14^ However, previous studies evaluate older generation femoral implants without accommodating trochlear grooves and lack evaluation of the degree of OA in the PFJ. In addition, a certain percentage of the anterior knee pain, which surgeons previously attributed to unresurfaced patellae in historical studies, was likely unrecognized flexion instability, which has identical presenting symptoms, and was an unknown clinical entity at the time. This likely explains the observed and often quoted fact that up to 50% of secondary resurfacing of unresurfaced patellae in TKA failed to alleviate anterior knee pain symptoms.^15,16,17,18,19^ Furthermore, a paucity of data exists evaluating the effect of patellofemoral OA on patient-reported outcomes particularly for unresurfaced patella using these modern “patella-friendly” implants.^20^

The goal of this study was to determine the effect of preoperative patellofemoral OA severity on postoperative patient-reported outcome measures (PROMs) after primary TKA with an unresurfaced patella using contemporary implants. The null hypothesis of the study was that there would be no significant effect of patellofemoral OA severity on PROMs.

## Methods

With institutional review board approval, a retrospective review was conducted on 206 primary TKAs without patellar resurfacing performed by a single surgeon from November 2013 to December 2019. Thirteen cases were excluded: hybrid cementation technique (3), poor patella bone quality (2), patella osteonecrosis (1), nonstandard implant used: universal baseplate or posterior-stabilized (2), and patients who required re-operations unrelated to the patella (5), leaving a sample size of 193 available for analysis.

Modern perioperative and rehabilitation protocols were used in all cases with one of two modern implant systems: cruciate-retaining or cruciate-substituting with an anterior lip insert (Implant A; Triathlon, Stryker Orthopaedics) or an ultracongruent-bearing (Implant B; EMPOWR 3D, DJO Surgical). The decision for leaving the patella unresurfaced was determined by the senior surgeon. General indications for leaving the patella unresurfaced were consistent with “selective patella resurfacing” and include central congruent tracking, joint space preservation radiographically, and ≤ grade 3 patellar chondral damage. Patella osteophytes were removed with a rongeur, and release of the lateral patella retinaculum with resection of the lateral-most portion of the lateral facet was performed.

Patient charts were reviewed in the electronic medical record to collect potential confounding variables for patient outcomes. Data collected included demographics of age, body mass index, sex, and the American Society of Anesthesiologists Physical Status (ASA-PS) classification; surgical details of tourniquet use, postoperative drain use, implant fixation, implant type, posterior cruciate ligament status; and preoperative comorbidities of rheumatoid arthritis, psoriatic arthritis, fibromyalgia, systemic lupus erythematosus, any lumbar-specific spine pathology, depression, and preoperative opioid usage.

Radiographic measurements included preoperative and postoperative tibiofemoral angle and patellar tilt according to the Knee Society Radiographic Evaluation System^21^ performed by one rater. In addition, the medial and lateral facets of the patella were radiographically assessed by one rater to evaluate the extent of patellofemoral OA using the Kellgren-Lawrence (KL) grading system^22^ and the Osteoarthritis Research Society International (OARSI) classification,^23^ as previously described in the literature.^24^ The OARSI system was subdivided into assessment of marginal osteophyte grades and joint space narrowing grades for both medial and lateral patellar facets. Patients were grouped for statistical analysis by KL grades of zero or 1 versus 2 or more because a KL score of two or more was thought to indicate clear evidence of radiographic arthritic changes. Similarly, patients were grouped by OARSI marginal osteophyte grades of zero or 1 versus 2 or three and OARSI joint space narrowing grades of zero versus 1 or more. Furthermore, arthritic changes of the patella and trochlea were graded and documented in the surgical note using the Outerbridge classification^25^ based on intraoperative findings. Patients were grouped for analysis by Outerbridge scores of zero or 1 versus 2 or more.

### Outcomes

PROMs used in this study were prospectively collected preoperatively and at a minimum 1-year follow-up during routine clinic visits or by telephone interview. The PROMs collected included the UCLA activity level^26,27^; components of the modern Knee Society Score (KSS)^28^ related to pain with level walking, pain while climbing stairs, and the question “Does your knee feel normal?”; the Knee Injury and Osteoarthritis Outcome Score for Joint Replacement^29^; and a global satisfaction question “What is your current level of satisfaction with your knee replacement?” Answers ranged on a five-point scale from very satisfied to very dissatisfied.

The minimum 1-year follow-up for PROM data was surgically defined as ≥11 months from the date of surgery as patients frequently have 12-month postoperative visits that are not exactly 12 months after surgery because of personal schedules, life events, travel, etc. In addition, previous contemporary TKA outcome studies have demonstrated no difference in PROM values between 12 and 24 months.^30-33^ Therefore, peer-reviewed literature now accepts 1-year follow-up as the essential minimum clinical follow-up duration for PROMs and other functional measures rather than the historical 2-year follow-up that was used specifically for survivorship outcome measures.

### Statistical Analysis

All statistical analysis was conducted in Minitab 19. Outliers were assessed with a form of the Dixon ratio test dependent on the sample size. Univariate analysis was conducted using a two-sample Student *t*-test for continuous outcomes and a chi square test to evaluate independence among categorical outcomes, with a Fisher *P* value reported for 2 × 2 contingency tables. Multivariate statistical modeling was used to evaluate the association of all collected variables with PROMs. Binary logistic regression was used to discriminate between binary categorical outcomes based on potential predictor variables, and linear regression models were used to evaluate continuous outcome variables. First, a backward selection method was used, including variables from univariate analysis with *P* ≤ 0.200. Second, a forward selection method with Bayesian information criterion was used with all variables included (listed in Tables 1–3). The final models used the selection method that provided an optimized model fit. Two-way interactions were evaluated within the multivariate models. Multicollinearity among predictors measured by a variance inflation factor was low for all main effects in the final models (variance inflation factor ≤ 1.13). Statistical means are reported as mean ± SD. A significance level of 0.05 was used for final multivariate models.

**Table 1:** Cohort Characteristics

Variable	N	Mean	SD	Min	Max
Age, yr	193	60.6	10.7	34.7	80.1
BMI, kg/m^2^	193	35.5	7.7	20.4	56.4
Sex, % female	52.9% (102/193)	—	—	—	—
ASA-PS, % III or IV	56.5% (109/193)	—	—	—	—
Fibromyalgia	2.6% (5/193)	—	—	—	—
SLE	1.6% (3/193)	—	—	—	—
RA	2.1% (4/193)	—	—	—	—
PA	1.0% (2/193)	—	—	—	—
Depression	16.6% (32/193)	—	—	—	—
Lumbar spine pathology	11.92% (23/193)	—	—	—	—
Preoperative narcotic usage	15.5% (30/193)	—	—	—	—
Tourniquet use	13.5% (26/193)	—	—	—	—
Drain use	17.6% (34/193)	—	—	—	—
PCL status^a^, % preserved|partial release|full release	31.3%|12.0%|56.8%	—	—	—	—
Fixation type, % cemented|noncemented	38.9%|61.1%	—	—	—	—
Implant type, % implant A|implant B	20.7%|79.3%	—	—	—	—

ASA = American Society of Anesthesiologists, BMI = body mass index, PA = psoriatic arthritis, RA = rheumatoid arthritis, SLE = systemic lupus erythematosus, PCL = posterior cruciate ligament,UCLA = University of California Los Angeles

a One case missing PCL status from the surgical note, N = 192.

**Table 2:** Radiographic Measurements

Variable	N	Mean	SD	Minimum	Maximum
Preoperative patellar tilt, degrees	193	3.11	2.65	0.00	13.00
Postoperative patellar tilt, degrees	193	3.06	2.73	0.00	12.00
Change in patellar tilt (preoperative to postoperative)	193	−0.06	3.25	−7.00	8.00
Preoperative tibiofemoral alignment, degrees + varus	193	−0.34	5.36	−16.00	9.00
Postoperative tibiofemoral alignment, degrees + varus	193	−4.79	2.29	−10.00	6.00
Change in tibiofemoral alignment, degrees	193	−4.44	5.36	−15.00	8.00

**Table 3:** OA Severity Ratings

Kellgren-Lawrence OA Severity	0	1	2	3	4
Medial patella	15.6%	29.2%	33.9%	18.8%	2.6%
Lateral patella	5.7%	31.8%	44.8%	14.6%	3.1%
OARSI marginal osteophyte	0	1	2	3	
Medial patella	41.5%	24.9%	21.8%	11.9%	
Lateral patella	33.7%	44.6%	11.9%	9.8%	
OARSI joint space narrowing	0	1	2	3	
Medial PFJ	67.9%	22.8%	7.3%	2.1%	
Lateral PFJ	56.5%	34.2%	8.3%	1.0%	
Intraoperative Outerbridge chondral damage	0	1	2	3	4
Overall PFJ	1.6%	24.1%	57.6%	13.1%	3.7%

OA = osteoarthritis, OARSI = Osteoarthritis Research Society International, PFJ = patellofemoral joint

Statistical power was 85.5% using a significant mean difference of 2.0 and assumed SD of 3.0, with the lowest sample size for analysis groups in this study of 42 at a significance level of 0.05.

### Source of Funding

This study received no external funding.

## Results

A total of 193 cases were available for analysis. The mean follow-up was 19.6 ± 9.5 months (range 11.3 to 84.8 months). The cohort comprised 52.9% female with a mean age and body mass index of 60.6 ± 10.7 years and 35.5 ± 7.7 kg/m^2^, respectively. All cohort characteristics are described in Table 1. Statistical summaries of radiographic measurements and OA severity gradings are described in Tables 2 and 3, respectively. All mean PROM scores significantly improved for the cohort preoperatively to a minimum of 1-year follow-up (Table 4; *P* ≤ 0.001).

**Table 4:** Overall Patient-reported Outcomes

Variable	N	Mean	SD	Min	Max
Preoperative UCLA activity level	184	4.7	2.1	1.0	10.0
Minimum 1-yr UCLA activity level	145	6.5	1.9	2.0	10.0
Change in UCLA activity level	140	1.8	2.2	−6.0	7.0
Preoperative KSS pain with level walking	180	6.2	2.2	0.0	10.0
Minimum 1-yr KSS pain with level walking	143	1.2	2.2	0.0	9.0
Change in KSS pain with level walking	138	−5.0	3.1	−10.0	6.0
Preoperative KSS pain while climbing stairs	180	7.9	1.9	2.0	10.0
Minimum 1-yr KSS pain while climbing stairs	143	1.7	2.6	0.0	9.0
Change in KSS pain while climbing stairs	138	−6.3	3.2	−10.0	4.0
Preoperative KOOS JR total	168	46.9	13.1	0.0	84.6
Minimum 1-yr KOOS JR total	138	82.0	16.4	34.2	100.0
Change in KOOS JR total	126	35.6	19.3	−29.1	84.1
Preoperative KSS knee normal % always feels normal	1.1% (2/180)	—	—	—	—
Minimum 1-year KSS knee normal % always feels normal	41.6% (59/142)	—	—	—	—
Minimum 1-year global satisfaction % very satisfied or satisfied	83.2% (119/143)	—	—	—	—

KOOS JR = Knee Injury and Osteoarthritis Outcome Score for Joint Replacement, KSS = Knee Society Score

Change = mean change from preoperative to minimum 1-year data.

### UCLA Activity Level

After multivariate analysis, the noncemented fixation type (β = 0.85, β-standard error [SE] 0.30, *P* = 0.005) and patients without depression (β = 0.96, SE 0.40, *P* = 0.016) were significant main effects of higher UCLA activity level at minimum 1-year follow-up. However, no predictors were found for increased improvement in the UCLA activity level from preoperative to minimum 1-year follow-up. KL OA severity grades, OARSI atlas grades, and Outerbridge scores were not predictive of any UCLA activity level scores in multivariate analysis.

### Knee Society Score Pain With Level Walking

**Figure 1:**
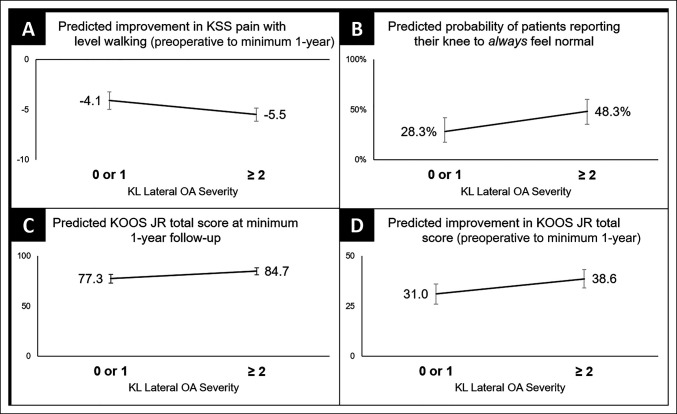
Worse preoperative KL lateral OA grade (≥2) was predictive of superior outcome scores compared to grades 0 or 1 (**A**–**D**), however a reasonable amount of overlap in the confidence intervals between groups was present. KL = Kellgren-Lawrence; OA = osteoarthritis

After multivariate analysis, there were no main effects for lower KSS pain with level walking scores at the minimum 1-year follow-up. However, a KL lateral OA grade of two or more was a significant predictor of greater improvement for KSS pain with level walking from preoperative to minimum 1-year follow-up (Figure 1, A; β = −1.39, SE 0.55, *P* = 0.013). KL medial OA grade, OARSI atlas grades, and Outerbridge classification were not predictive of any KSS pain with level walking scores in multivariate analysis.

### Knee Society Score Pain While Climbing Stairs or Inclines

After multivariate analysis, there were no main effects for lower KSS pain while climbing stairs scores at minimum 1-year follow-up or for the change from preoperative to minimum 1-year follow-up. Specifically, KL OA grades, OARSI atlas grades, and Outerbridge classification were not predictive of any KSS pain while climbing stairs scores in multivariate analysis.

### Knee Society Score—Does This Knee Feel Normal?

After multivariate analysis, only a KL lateral OA grade of two or more (OR 3.12; *P* = 0.005) and an OARSI lateral marginal osteophyte grade of 0 or 1 (OR 2.72, *P* = 0.030) were the main effects of knees “always feeling normal” at minimum 1-year follow-up (Figure 1, B). KL medial OA grade, OARSI medial marginal osteophyte grade, OARSI joint space narrowing grades, and Outerbridge classification were not predictive of any KSS scores related to knees feeling normal in multivariate analysis.

### Knee Injury and Osteoarthritis Outcome Score for Joint Replacement Total

After multivariate analysis, only a KL lateral OA grade of two or more was a main effect of higher KOOS JR total score at minimum 1-year follow-up (Figure 1, C; β = 7.42, SE 2.83, *P* = 0.010). Again, only a KL lateral OA grade of two or more was a predictor for greater improvement in the KOOS JR total score from preoperative to minimum 1-year follow-up (Figure 1, D; β = 7.57, SE 3.43, *P* = 0.029). Again, KL medial OA grade, OARSI atlas grades, and Outerbridge classification were not predictive of any KOOS JR total scores in multivariate analysis.

### Global Satisfaction

After multivariate analysis, there were no main effects for higher global satisfaction scores at minimum 1-year follow-up. Particularly, KL OA grades, OARSI atlas grades, and Outerbridge classification were not predictive of greater satisfaction scores in multivariate analysis. All multivariate results are summarized in Table 5.

**Table 5:** Summary of Main Study Findings From Multivariate Analysis

Outcome	Significant Main Effect(s)	*P*	VIF	β SE or Odds Ratio, 95% CI
Change in KSS pain with level walking	KL lateral OA grade ≥2	**0.013**	1.00	β = −1.39 SE 0.55
KSS knee normal score “knees feeling always normal”	KL lateral OA grade ≥2	**0.005**	1.13	OR 3.12 1.42–6.87
	OARSI lateral marginal osteophyte grade ≤1	**0.030**	1.13	OR 2.72 1.10–6.71
KOOS JR total	KL lateral OA grade ≥2	**0.010**	1.00	β = 7.42 SE 2.83
Change in KOOS JR total score	KL lateral OA grade ≥2	**0.029**	1.00	β = 7.57 SE 3.43

CI = confidence interval, KL = Kellgren-Lawrence, KSS = Knee Society Score, OA = osteoarthritis, OARSI = Osteoarthritis Research Society International, OR = odds ratio, SE = standard error, VIF = variance inflation factor, β = coefficient of linear regression model

Bold *P* values indicate statistical significance.

### Re-operation Surgery

A total of 2.4% of the cases (5/206) required a re-operation surgery due to any reason at a mean of 17.8 months (range 1.2 to 66.6 months). No cases required revision due to progressive patellar OA, anterior knee pain, or extensor mechanism complications after primary TKA. One femoral implant was revised for aseptic loosening, one case required revision because of a nickel allergy and metal hypersensitivity, and another case was revised because of tibial insert polyethylene wear. One case required re-operation because of quadriceps and arthrotomy disruption with implant retention. One case required an irrigation and débridement with implant retention because of a complex primary wound closure.

## Discussion

The results of this study do not show a deleterious effect of increased radiographic or intraoperative arthritic changes on outcomes in the setting of an unresurfaced patella after primary TKA with the use of modern implant systems. In fact, when significant differences in the postoperative outcomes were detected, the patients with greater levels of radiographic arthritis demonstrated superior outcomes. Specifically, multivariate analysis showed a positive correlation of KL grade ≥2 in the lateral facet of the PFJ with superior absolute and delta change in the KOOS JR total score, delta change in KSS pain with level walking, and a greater likelihood of a patient reporting their knee to “always feel normal.” Although these findings were statistically significant in multivariate analysis, confidence intervals did overlap calling into question the clinical and statistical significance of these findings. Interestingly, Outerbridge scores did not correlate with any PROMs in multivariate analysis. In addition, only decreased OARSI lateral marginal osteophyte grade was associated with a greater likelihood of a patient reporting their knee to always feel normal. All other OARSI atlas grades (marginal osteophyte and joint space narrowing) did not correlate with any PROMs in multivariate analysis. Furthermore, no cases were revised because of patella-related issues, which may be due, in part, to the more “patella-friendly” femoral implants used in this study. These implants are designed with more anatomically shaped trochlear grooves and slightly elevated lateral anterior femoral flanges to help correct the patella to a more neutral alignment and therefore possibly less complications postoperatively.

This study provides important insight into the relationship between preoperative and intraoperative observation of arthritic changes and postoperative outcomes after primary TKA with unresurfaced patellae. Previous literature has shown patellar resurfacing to be associated with decreased anterior knee pain and lower re-operation rates.^1^ However, studies have examined the outcomes of selective patellar resurfacing with good results. For example, Kim et al^34^ showed good results compared with routine resurfacing of the patella when conducting selective resurfacing based on the presence of articular cartilage without eburnation, satisfactory tracking of the native patella, no inflammatory or crystalline arthritis, and normal patellar shape. Similarly, Maradit-Kremers et al^35^ evaluated 402 TKAs with selectively unresurfaced patellae using similar criteria as that in the study of Kim et al^34^ within a cohort of 21,371 TKAs and did not find a significantly increased risk of patella-related complications or re-operations when controlling for other factors. These results indicate that patellar resurfacing may not provide significant clinical benefit in certain patient cohorts.

Although proponents of selective patellar resurfacing commonly use the shape of the patella and quality of the articular cartilage as factors to consider when deciding to resurface the patella intraoperatively, it is unclear how the presence and magnitude of arthritic changes in the PFJ affect these postoperative outcomes. Previously, Rodríguez-Merchán et al^14^ conducted a study in 500 TKA patients divided into groups based on the Outerbridge classification of their patella and then randomized to either resurfacing or nonresurfacing. Patients with grade 1 to 3 were placed in group A, and patients with grade 4 were placed in group B. At minimum 5-year follow-up, the authors found that 11.6% of the patients in group B required secondary patella resurfacing as compared to 0.6% in group A. Although this study solely evaluated re-operation rates and the implant used for all procedures was an older generation design, these results indicate a potential effect of end-stage patellar arthritis on outcomes with unresurfaced patellae. However, this study evaluated older generation implants that are not as “patella-friendly” compared to contemporary designs. In addition, a more granular breakdown of Outerbridge grades 1 to 3 was not evaluated; furthermore, no patient-reported outcomes were reported in this study. Another study by Cho et al^20^ evaluated clinical and radiographic outcomes comparing patellofemoral arthritis by the Iwano classification system and found no differences, which corroborate findings of this study.

The null hypothesis was partially rejected because there was some evidence to suggest worse preoperative PFJ OA in the lateral facet by the Kellgren-Lawrence grading system correlated with superior knee-specific PROMs. However, this was the only variable across all outcomes that was consistently a predictor of PROMs and may simply reflect the positive effect of increased radiographic arthritis on postoperative outcomes. For example, Polkowski et al^36^ demonstrated a higher risk of postoperative pain and dissatisfaction when preoperative radiographs were indicative of minimal degenerative changes. Similarly, van de Water et al^37^ clearly showed improved postoperative pain and function as KL scores increased. Although this study did not evaluate the severity of tibiofemoral OA in the cohort, it is possible that patients with increased radiographic OA in the PFJ were more likely to have more advanced arthritic changes throughout the knee and were predisposed for a better outcome.

This study should be considered in the context of its limitations. First, the retrospective nature of this study may introduce inherent bias; however, all outcomes of this study were prospectively collected. A randomized control trial with multiple surgeons would be ideal to truly remove all potential biases. Second, the decision to leave the patella unresurfaced during TKA was intraoperatively determined by the treating surgeon, which could create the potential for selection bias. Third, this study only had one rater for radiographic measurements and ratings. However, the rater had extensive training from the senior surgeon and research team before formal data collection. Fourth, comparison groups within each implant type with resurfaced patella were not evaluated. This study also had strengths related to all patients being treated by one surgeon with contemporary “patella-friendly” implants and modern perioperative and rehabilitation protocols, limiting the variability within the cohort but may limit external validity of findings. In addition, this study evaluated PROMs, which provide a more scientifically rigorous investigation compared to the use of re-operation rates alone.

In summary, worse preoperative OA severity in the lateral patellar facet, graded with the KL system, predicted superior knee-specific PROMs in patients with unresurfaced patellae after contemporary TKA. This observation highlights the minimal contribution of radiographic or intraoperative patella OA to knee function after TKA for tibiofemoral disease. Additional research is warranted to delineate selective patella resurfacing criteria for optimal primary TKA outcomes.

## References

[1] Meneghini RM: Should the patella Be resurfaced in primary total knee arthroplasty? An evidence-based analysis. J Arthroplasty 2008;23(7 suppl):11-14. 18701250 10.1016/j.arth.2008.06.009

[2] Schindler OS: The controversy of patellar resurfacing in total knee arthroplasty: Ibisne in medio tutissimus? Knee Surg Sports Traumatol Arthrosc 2012;20:1227-1244. 22484417 10.1007/s00167-012-1985-7 PMC3378836

[3] Abdel MP Parratte S Budhiparama NC: The patella in total knee arthroplasty: To resurface or not is the question. Curr Rev Musculoskelet Med 2014;7:117-124. 24706154 10.1007/s12178-014-9212-4 PMC4092199

[4] McConaghy K Derr T Molloy RM Klika AK Kurtz S Piuzzi NS: Patellar management during total knee arthroplasty: A review. EFORT Open Rev 2021;6:861-871. 34760286 10.1302/2058-5241.6.200156 PMC8559560

[5] AAOS. Ajrr Annual Report. In. 2021. https://connect.ajrr.net/2021-ajrr-annual-report. Accessed December 20, 2021.

[6] Zmistowski BM Fillingham YA Salmons HI Ward DT Good RP Lonner JH: Routine patellar resurfacing during total knee arthroplasty is not cost-effective in patients without patellar arthritis. J Arthroplasty 2019;34:1963-1968. 31104838 10.1016/j.arth.2019.04.040

[7] Meding JB Fish MD Berend ME Ritter MA Keating EM: Predicting patellar failure after total knee arthroplasty. Clin Orthop Relat Res 2008;466:2769-2774. 18712456 10.1007/s11999-008-0417-y PMC2565015

[8] Pagnano MW Trousdale RT: Asymmetric patella resurfacing in total knee arthroplasty. Am J Knee Surg 2000;13:228-233. 11269543

[9] O'Brien S Spence DJ Ogonda LO Beverland DE: Lcs mobile bearing total knee arthroplasty without patellar resurfacing. Does the unresurfaced patella affect outcome? Survivorship at a minimum 10-year follow-up. Knee 2012;19:335-338. 21856160 10.1016/j.knee.2011.07.002

[10] Ma HM Lu YC Kwok TG Ho FY Huang CY Huang CH: The effect of the design of the femoral component on the conformity of the patellofemoral joint in total knee replacement. J Bone Joint Surg Br 2007;89:408-412. 17356162 10.1302/0301-620X.89B3.18276

[11] Pilling RW Moulder E Allgar V Messner J Sun Z Mohsen A: Patellar resurfacing in primary total knee replacement: A meta-analysis. J Bone Joint Surg Am 2012;94:2270-2278. 23318618 10.2106/JBJS.K.01257

[12] Clements WJ Miller L Whitehouse SL Graves SE Ryan P Crawford RW: Early outcomes of patella resurfacing in total knee arthroplasty. Acta Orthop 2010;81:108-113. 19968604 10.3109/17453670903413145 PMC2856213

[13] Lygre SH Espehaug B Havelin LI Vollset SE Furnes O: Failure of total knee arthroplasty with or without patella resurfacing. Acta Orthop 2011;82:282-292. 21619502 10.3109/17453674.2011.570672 PMC3235305

[14] Rodriguez-Merchan EC Gomez-Cardero P: The outerbridge classification predicts the need for patellar resurfacing in TKA. Clin Orthop Relat Res 2010;468:1254-1257. 19844770 10.1007/s11999-009-1123-0 PMC2853678

[15] Campbell DG Mintz AD Stevenson TM: Early patellofemoral revision following total knee arthroplasty. J Arthroplasty 1995;10:287-291. 7673906 10.1016/s0883-5403(05)80176-7

[16] Karnezis IA Vossinakis IC Rex C Fragkiadakis EG Newman JH: Secondary patellar resurfacing in total knee arthroplasty: Results of multivariate analysis in two case-matched groups. J Arthroplasty 2003;18:993-998. 14658103 10.1016/s0883-5403(03)00286-9

[17] Muoneke HE Khan AM Giannikas KA Hägglund E Dunningham TH: Secondary resurfacing of the patella for persistent anterior knee pain after primary knee arthroplasty. J Bone Joint Surg Br 2003;85:675-678. 12892189

[18] Khatod M Codsi M Bierbaum B: Results of resurfacing a native patella in patients with a painful total knee arthroplasty. J Knee Surg 2004;17:151-155. 15366270 10.1055/s-0030-1248214

[19] Mockford BJ Beverland DE: Secondary resurfacing of the patella in mobile-bearing total knee arthroplasty. J Arthroplasty 2005;20:898-902. 16230242 10.1016/j.arth.2005.02.009

[20] Cho WJ Bin SI Kim JM Lee BS Sohn DW Kwon YH: Total knee arthroplasty with patellar retention: The severity of patellofemoral osteoarthritis did not affect the clinical and radiographic outcomes. J Arthroplasty 2018;33:2136-2140. 29576487 10.1016/j.arth.2018.02.075

[21] Meneghini RM Mont MA Backstein DB Bourne RB Dennis DA Scuderi GR: Development of a modern knee society radiographic evaluation system and methodology for total knee arthroplasty. J Arthroplasty 2015;30:2311-2314. 26122112 10.1016/j.arth.2015.05.049

[22] Kellgren JH Lawrence JS: Radiological assessment of osteo-arthrosis. Ann Rheum Dis 1957;16:494-502. 13498604 10.1136/ard.16.4.494 PMC1006995

[23] Altman RD Gold GE: Atlas of individual radiographic features in osteoarthritis. Revised Osteoarthritis Cartilage 2007;15(suppl A):A1-A56. 17320422 10.1016/j.joca.2006.11.009

[24] Deckard ER Jansen K Ziemba-Davis M Sonn KA Meneghini RM: Does patellofemoral disease affect outcomes in contemporary medial fixed-bearing unicompartmental knee arthroplasty? J Arthroplasty 2020;35:2009-2015. 32234327 10.1016/j.arth.2020.03.007

[25] Slattery C Kweon CY: Classifications in brief: Outerbridge classification of chondral lesions. Clin Orthop Relat Res 2018;476:2101-2104. 29533246 10.1007/s11999.0000000000000255 PMC6259817

[26] Zahiri CA Schmalzried TP Szuszczewicz ES Amstutz HC: Assessing activity in joint replacement patients. J Arthroplasty 1998;13:890-895. 9880181 10.1016/s0883-5403(98)90195-4

[27] Naal FD Impellizzeri FM Leunig M: Which is the best activity rating scale for patients undergoing total joint arthroplasty? Clin Orthop Relat Res 2009;467:958-965. 18587624 10.1007/s11999-008-0358-5 PMC2650053

[28] Scuderi GR Sikorskii A Bourne RB Lonner JH Benjamin JB Noble PC: The knee society short form reduces respondent burden in the assessment of patient-reported outcomes. Clin Orthop Relat Res 2016;474:134-142. 26047645 10.1007/s11999-015-4370-2 PMC4686526

[29] Lyman S Lee YY Franklin PD Li W Cross MB Padgett DE: Validation of the KOOS, JR: A short-form knee arthroplasty outcomes survey. Clin Orthop Relat Res 2016;474:1461-1471. 26926773 10.1007/s11999-016-4719-1 PMC4868168

[30] Ramkumar PN Navarro SM Haeberle HS Ng M Piuzzi NS Spindler KP: No difference in outcomes 12 and 24 Months after lower extremity total joint arthroplasty: A systematic review and meta-analysis. J Arthroplasty 2018;33:2322-2329. 29567000 10.1016/j.arth.2018.02.056

[31] Piuzzi NS: Patient-reported outcomes at 1 and 2 years after total Hip and knee arthroplasty: What is the minimum required follow-up? Arch Orthop Trauma Surg 2021. 10.1007/s00402-021-03819-x 33606086

[32] Samuelsson K Magnussen RA Alentorn-Geli E : Equivalent knee Injury and osteoarthritis outcome scores 12 and 24 Months after anterior cruciate ligament reconstruction: Results from the Swedish national knee ligament register. Am J Sports Med 2017;45:2085-2091. 28714793 10.1177/0363546517702871

[33] Seetharam A Deckard E Ziemba-Davis M Meneghini R: The AAHKS clinical research award: Are minimum two-year proms necessary for accurate assessment of patient outcomes after primary TKA? J Arthroplasty 2021. 10.1016/j.arth.2022.02.016 35151810

[34] Kim BS Reitman RD Schai PA Scott RD: Selective patellar nonresurfacing in total knee arthroplasty. 10 year results. Clin Orthop Relat Res 1999;367:81-88. 10546601

[35] Maradit-Kremers H Haque OJ Kremers WK : Is selectively not resurfacing the patella an acceptable practice in primary total knee arthroplasty? J Arthroplasty 2017;32:1143-1147. 27876254 10.1016/j.arth.2016.10.014

[36] Polkowski GG II Ruh EL Barrack TN Nunley RM Barrack RL: Is pain and dissatisfaction after TKA related to early-grade preoperative osteoarthritis? Clin Orthop Relat Res 2013;471:162-168. 22923158 10.1007/s11999-012-2465-6 PMC3528890

[37] van de Water RB Leichtenberg CS Nelissen R : Preoperative radiographic osteoarthritis severity modifies the effect of preoperative pain on pain/function after total knee arthroplasty: Results at 1 and 2 years postoperatively. J Bone Joint Surg Am 2019;101:879-887. 31094979 10.2106/JBJS.18.00642

